# Primary Circulating Prostate Cells Are Not Detected in Men with Low Grade Small Volume Prostate Cancer

**DOI:** 10.1155/2014/612674

**Published:** 2014-08-19

**Authors:** Nigel P. Murray, Eduardo Reyes, Cynthia Fuentealba, Nelson Orellana, Omar Jacob

**Affiliations:** ^1^Hospital Carabineros of Chile, Nunoa, 7770199 Santiago, Chile; ^2^Circulating Tumor Cell Unit, Faculty of Medicine, University Mayor, Las Condes, 7550224 Santiago, Chile; ^3^Institute of Bio-Oncology, Providencia, 7500710 Santiago, Chile; ^4^Faculty of Medicine, Diego Portales University, Manuel Rodríguez Sur 415, 8370179 Santiago, Chile

## Abstract

*Objective*. To determine if primary circulating prostate cells (CPCs) are found in all men with prostate cancer. *Methods and Patients*. A prospective study, to analyze all men with an elevated PSA between 4.0 and 10.0 ng/mL undergoing initial biopsy. Primary CPCs were obtained by differential gel centrifugation and detected using standard immunocytochemistry using anti-PSA; positive samples underwent a second process with anti-P504S. A malignant primary CPC was defined as PSA (+) P504S (+) and a test positive if 1 cell/4 mL was detected. Biopsy results were registered as cancer/no-cancer, number of cores positive, and percent infiltration of the cores. *Results*. 328/1123 (29.2%) of the study population had prostate cancer diagnosed on initial biopsy, and 42/328 (12.8%) were negative for primary CPCs. CPC negative men were significantly older, and had lower PSA levels, lower Gleason scores, and fewer positive cores and with infiltration by the cancer. 38/42 (91%) of CPC negative men complied with the criteria for active surveillance in comparison with 34/286 (12%) of CPC positive men. *Conclusions*. Using primary CPC detection as a sequential test to select men with an elevated PSA for biopsy, the risk of missing clinically significant prostate cancer is minimal when the patient is primary CPC negative; less than 0.5% of all primary CPC negative men had a clinically significant prostate cancer.

## 1. Introduction

Prostate cancer is the second most common cancer and second cause of cancer death in Chilean men [1].

Prostate specific antigen (PSA) is the most accurate serum marker for prostate and the only biomarker routinely used for the early detection of prostate cancer. Although PSA is highly specific for prostate, an elevated level is not specific for prostate cancer, being increased in benign pathologies [2, 3]. Consequently, approximately 70% of men with an increased serum PSA, defined as >4.0 ng/mL, do not have prostate cancer [4] and thus undergo unnecessary prostate biopsies. A PSA cutoff of 4.0 ng/mL is currently used to select men for prostate biopsy; however, this misses many cancers and it has been suggested that lowering the cutoff to 2.6 ng/mL will detect small but clinically significant cancers [5]. The Prostate Cancer Prevention Trial [6] reported that 39.2% of men with a PSA 2.1–3.0 ng/mL, 27.7% of men with a PSA 1.1–2.0 ng/mL, and 1.3% of men with a PSA <1.0 ng/mL had end of trial prostate biopsies with foci of adenocarcinoma. In other words 38% of men with prostate cancer have a PSA <4.0 ng/mL and 70% of men with a PSA >4.0 ng/mL do not have cancer. The subject is further complicated by the high prevalence of prostate cancer detected at autopsy [7], the high frequency of positive prostate biopsies in men with a normal digital rectal examination (DRE) and PSA <4.0 ng/mL [8], the contrast between the incidence and mortality rates for prostate cancer, and the need to treat an estimated 37 men with screened detected prostate cancer to prevent one prostate cancer death [9, 10]. To achieve a relative mortality reduction of 40% by screening for prostate cancer [11], 50% of screened detected prostate cancers may be overtreated [12]. Thus the search for new biomarkers to improve the diagnostic yield is needed. This is especially so as the risks of prostate biopsy are not insignificant; Rietbergen et al. [13], in a study of 5,802 patients undergoing transrectal prostate biopsy, reported an incidence of complications of 0.5% hospitalizations, 2.1% rectal hemorrhage, 2.3% fever, and 7.2% persistent hematuria.

The use of primary malignant CPC (mCPC) detection as a sequential test for deciding the need for prostate biopsy may resolve in part some of these problems. In the study ProTECT of 228 patients undergoing first biopsy where 28.5% of patients had cancer diagnosed, the detection of primary mCPCs and the association with a positive biopsy had a sensitivity of 86.2% and specificity of 90.8%, with a positive predictive value of 78.9% and negative predictive value of 97.1% [14]. The use of the detection of primary mCPCs as a sequential test to select men, with suspicion of prostate cancer for an elevated PSA, to determine the need for prostate biopsy raised concern of the possibility of missing clinically significant prostate cancer. We present the results of 328 men diagnosed with prostate cancer as a result of a screening program and compare the results of primary mCPC detection with the biopsy Gleason score, percent of infiltration of the samples by cancer, and the number of positive cores. We used the same method of mCPC detection as described in the ProTECT study.

## 2. Methods and Patients

After ethical committee approval of the study for the use of primary mCPC detection, a prospective study was carried out. All patients attended in the Carabineros de Chile Health System and had a serum PSA >4.0 ng/mL and/or a digital rectal examination (DRE) suspicious of prostate cancer and were referred for prostate biopsy. Immediately before the biopsy 8 mL of venous blood was collected in EDTA (Beckinson-Vacutainer) and sent to a central laboratory. Patients were coded and clinical details of serum PSA, age, and biopsy results were collected.

### 2.1. Detection of mCPCs

Blood samples were maintained at 4°C and processed within 48 hours. The prostate biopsy and primary CPC detection were independently analyzed, with the evaluators being blinded to the clinical details and results of the biopsy or primary mCPC test.

Mononuclear cells were obtained by differential centrifugation using Histopaque 1,077 (Sigma-Aldrich), washed, and resuspended in 100 *μ*L of autologous plasma. 25 *μ*L aliquots were used to make slides (silanized, DAKO, USA), dried in air for 24 hours, and fixed in a solution of 70% ethanol, 5% formaldehyde, and 25% phosphate buffered saline, pH 7.4.

Primary mCPCs were detected using a monoclonal antibody directed against PSA and clone 28A4 (Novocastro Laboratory, UK) and identified using an alkaline phosphatase-anti-alkaline phosphatase based system (LSAB2, DAKO, USA), with new.-fushcin as the chromogen. Positive samples underwent a second process with anti-P504S clone 13H4 (DAKO, USA) and were identified with a peroxidase based system (LSAB2, DAKO, USA) with DAB (3,3′-diaminobenzidine tetrahydrochloride) as the chromogen.

**Figure 1:**
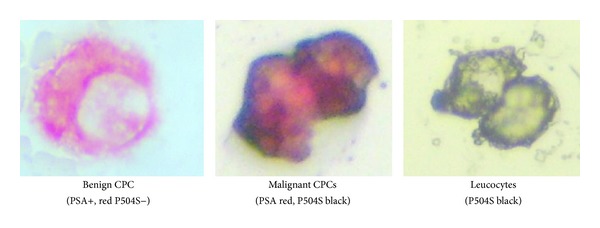
Photomicrography of CPCs.

A primary mCPC was defined according to the criteria of ISHAGE (International Society of Hematotherapy and Genetic Engineering) [15] and the expression of P504S according to the Consensus of the American Association of Pathologists [16]. A malignant primary CPC (mCPC) was defined as a cell that expressed PSA and P504S; a benign primary CPC (bCPC) expressed PSA but not P504S and leucocytes could be P504S positive or negative but did not express PSA (Figures 1(a)–1(c)). A test was considered positive when at least 1 cell was detected/4 mL blood. The test was classified as positive or negative for mCPC; the number of mCPCs/4 mL of blood was not used as a parameter in order to simplify the result of the test. Immunocytochemical staining of the slides was analyzed by one observer blinded to clinical and pathological details.

### 2.2. Pathological Analysis of the Biopsy

An ultrasound guided 12-core biopsy was taken according to standard recommendations; samples were fixed in formaldehyde and sent to the Pathology Service. 3 *μ*m paraffin embedded sections were cut, deparaffinized, and stained with H&E as per standard procedure. If cancer was detected, Gleason score, number of cores positive for cancer, and percent of infiltration were recorded. The prostate biopsy was analyzed by a single pathologist blinded to clinical details and results of the mCPC test. The ultrasound was used to guide the biopsy; not all reports had the prostate volume calculated or whether the prostate was homogeneous or heterogeneous. As it is not routine in Chile the use of prostatic ultrasound prebiopsy, we did not include this parameter in the analysis.

### 2.3. Comparison of Primary mCPC Status with Criteria NCCN and NICE for Active Observation as a Treatment Option for Prostate Cancer

Both the NCCN guidelines and NICE (UK) guidelines recommend as a treatment option active surveillance in men with the following criteria: clinical stage T1c or less, Gleason score ≤6, less than 3 cores positive for cancer, and less than 50% or 10 mm infiltration in any one core. The frequency of patients who were primary mCPC positive and negative was compared using these guidelines to evaluate the number of patients complying with the criteria for active surveillance. We used the 1994 criteria for AS and not those of Tosoian et al. [17] as not all patients had prostate volume registered in the ultrasound report.

### 2.4. Statistical Analysis

Descriptive statistics were used for demographic variables, expressed as mean and standard deviation in the case of continuous variables with a normal distribution. In case of an asymmetrical distribution the median and interquartile range (IQR) values were used. Noncontiguous variables were presented as frequencies. The Shapiro-Wilk test was used to determine a normal distribution. The Student *t*-test was used to compare continuous variables with a normal distribution, the Mann-Whitney test for ordinate and continuous variables with a nonnormal distribution, and Chi-squared for the differences in frequency. Statistical significance was defined as a *P* value less than 0.05 to all tests were two tailed. Analysis was performed using the Stata 11.0 program (StataCorp LP, College Station, Texas, USA).

### 2.5. Ethical Considerations

The study was directed with complete conformity with the principles of the Declaration of Helsinki and approval of the local ethical committees.

## 3. Results

A total of 328 men of a cohort of 1123 patients had a prostate biopsy positive for cancer, with an overall incidence of 29.2% of all biopsies. 42/328 (12.8%) of these men were negative for the detection of primary mCPCs. The clinic-pathological details are shown in Table 1. Men negative for primary mCPCs were significantly older and had lower serum PSA levels, lower Gleason scores, lower number of cores positive for prostate cancer, and cores less infiltrated with cancer. Of the 1123 patients, 90 (8%) were positive for mCPCs but had an initial prostate biopsy negative for cancer.

### 3.1. Number of Men Complying with the Criteria for Treatment with Active Surveillance

Comparing men mCPC negative with those mCPC positive using the Epstein criteria [18] for active surveillance, 38/42 (91%) of mCPC negative men compared with 34/286 (12%) (*P* < 0.0001) of mCPC positive men complied with the criteria for active surveillance of their prostate cancer (Table 2).

Four men in the CPC negative group did not comply with the criteria for active surveillance; their details are shown in Table 3. In patient number 3 it was an incidental finding, one microfocus of cancer. Patient number 1 had a cancer which needed treating and underwent radical prostatectomy as did Patients 2 and 4.

## 4. Discussion

Models of prostate cancer detection and estimates of progression suggest that 23–42% of screen detected prostate cancers are overtreated [19]. The introduction by Epstein et al. [18] of criteria to predict pathologically “insignificant” prostate cancer has been useful but there is caution about using it as the sole reference for making clinical decisions as many as 8% of these cancers were not organ confined based on postsurgical findings [20]. Other nonograms have been proposed and reviewed by Bastian et al. [21]. Active surveillance is considered the best option for patients with low risk cancers or a short life expectancy. Thus if there is a significant percent of men overtreated for their prostate cancer and active surveillance is an accepted treatment option, then the aim of the prostate biopsy in men with an elevated PSA is not to detect each and every prostate cancer but to detect those prostate cancers with the potential for causing harm. Men with clinically insignificant prostate cancers that were never destined to have symptoms or to affect their life expectancy may not benefit from knowing that they have the “disease.” The detection of clinically insignificant prostate cancer could be considered as an adverse effect of the prostate biopsy. As such, there is considerable anxiety and distress found in men undergoing active surveillance [22].

There are no directly relevant studies comparing immediate and delay biopsy in men with a raised PSA level. A number of observational studies have reported risk factors for high grade prostate cancer in men referred for biopsy, related to age, PSA, DRE result, prior prostate biopsy, black ethnicity, and prostate volume [23–25]. However, there are concerns over delaying a prostate biopsy because of the uncertainties of the natural history of untreated prostate cancer, the missed opportunity to detect and treat a curable cancer, or that due to delay in performing a biopsy the treatment of a larger or more aggressive cancer may lead to a more complex surgery with greater side effects.

The use of primary CPC detection to select men for prostate biopsy fails to detect men with CPC negative cancer. This represents approximately 5% of all primary CPC negative men and an elevated PSA of between 4.0 and 10.0 ng/mL [14]. This study suggests that, of these 5% of primary CPC negative men, 9% would have a prostate cancer complying with the guidelines for treatment or that approximately 99.5% of all primary CPC negative men would not have a prostate cancer needing treatment or in the majority of cases primary CPC negative having benign prostatic hyperplasia [14]. Thus from these results the concern of missing significant prostate cancer is minimal, much less than the 38% of prostate cancers missed when using a PSA level of 4.0 ng/mL as a cutoff point for recommending prostate biopsy [6]. An ongoing study of the followup of all primary CPC negative men with an elevated PSA is currently in progress.

We used the Epstein criteria to define active surveillance rather than those of Tosoian et al. [17]; men do not routinely undergo prostatic ultrasound as part of the prostate cancer screening program; thus the decision to refer the patient for prostate biopsy based on PSA and mCPC determinations would not include prostate volume determinations. Previously we reported the use of the number of CPCs/mL blood detected; however, the increase in specificity using a cutoff point of 4 cells/mL blood was minimal, 8%, with an important decrease in sensitivity [14].

There are two questions not answered by the study, which are currently part of an ongoing investigation. Firstly, mCPC positive men with a negative biopsy are these men at increased risk of prostate cancer, in that they have cancer but the biopsy failed to detect it; studies have shown that approximately 20% of men have cancer detected on repeated biopsy [26]. If follow-up studies show that these men do indeed have cancer, it may be advisable to repeat the biopsy earlier or a biopsy for saturation. Secondly, in mCPC negative men, we are currently repeating total and free PSA with mCPC testing on a 6-month basis; men who become positive, with an abnormal DRE or significant change in total PSA and/or free PSA, are referred for biopsy. It would be important to determine if a change from mCPC negative to positive was associated with a change in the clinic-pathological parameters to indicate active treatment. In Chile, few men choose to undergo active surveillance, preferring active treatment, as we have little data on this type of patient. It is important to emphasize that these are primary mCPCs and are not associated with the prognosis; most of these cells disappear after radical treatment; men who remain positive, secondary mCPC positive, have a higher frequency of biochemical failure [27].

One concern over the use of circulating tumor cell technologies is the discordant results achieved using different methods of detection. Using a dual PSA/prostate specific membrane antigen RT-PCR method Eschwège et al. [28] only found 37% of preoperative patients to be CPC positive. Davis et al. [29] found no association between CPC detection using the CellSearch system and the clinical parameters prior to radical prostatectomy or between men with local PC or controls. However, Stott et al. [30] found primary CPCs in 42% of patients with localized cancer; Fizazi et al. [31], using anti-BerEP-4 epithelial antigen combined with telomerase activity, detected primary CPCs in 79% of patients with localized cancer, a similar figure to that reported using this same methodology [14]. We believe that part of the difference documented is caused by the relatively high detection in control patients; one explication is that CPC can be found in men with prostatitis and benign hyperplasia; however, these CPCs are P504S negative [32]. We also designed the test using CPCs to produce a result considered as positive or negative; the fundamental question was “is there cancer and will it harm the patient?”; consequently we considered that the presence of single cell is sufficient to classify patients as positive or negative for cancer.

We also need to emphasize that this is a single institution study; thus the test needs to be used in other centers to determine the reproducibility of the test. This in part will be determined by the skill of the observer. Within our institution preanalytical variables are controlled and are limited; immunocytochemical staining can be automatically performed and thus the main variable is the observer. Observer variation could be minimized with adequate training but is a variable that needs to be considered. The inter-observer variation in reported Gleason scores has been a problem; recently published data on the clinical implications of interobserver variation showed an overall agreement of 80.7–89%, but this would lead to up to 10% of patients recommended for active observation would have received different treatments based on inter-observer variation [33].

## 5. Conclusions

The objective of this study was to address the concern of not detecting potentially harmful prostate cancer using the detection of P504S expressing primary CPCs. The results suggest that the majority of cancers that the test failed to detect, when used as a sequential screening test in men with a PSA level of between 4.0 and 10.0 ng/mL, are low grade small volume tumors which would comply with the criteria for active observation. Thus in primary CPC negative men the possibility of a harmful cancer being missed is minimal; in these men prostate biopsy could be avoided or delayed, thus not exposing the patient to the adverse effects of biopsy or the anxiety or distress of active observation.

## Figures and Tables

**Table 1:** Clinicopathological findings in mCPC positive and negative men with prostate cancer.

	CPC negative	CPC positive	
Age mean ± SD (years)	68.3 ± 8.9	65.5 ± 9.8	*P* < 0.001 (*t*-test)
PSA ng/mL median ± IQR	4.76 (4.50–6.43)	5.76 (4.76–9.67)	*P* < 0.001 (ANOVA)
Gleason score median ± IQR	4 (4-5)	6 (5–7)	*P* < 0.05 (ANOVA)
% infiltrated of the 12 cores, median ± IQR	5 (3–5)	30 (15–45)	*P* < 0.0001 (ANOVA)
Number of cores positive, median ± IQR	1 (1-2)	4 (3–7)	*P* < 0.001 (ANOVA)

IQR: interquartile range, CPC: circulating prostate cell.

**Table 2:** Frequency of mCPC negative and positive men complying with Epstein criteria for active surveillance.

	CPC negative (*N* = 42)	CPC positive (*N* = 286)	Chi-squared
Gleason ≤ 6	41 (98%)	98 (34%)	*P* < 0.0001
<3 cores positive	40 (95%)	60 (21%)	*P* < 0.0001
<50% infiltration in 1 core	42 (100%)	206 (72%)	*P* = 0.0002
All 3 criteria	38 (91%)	34 (12%)	*P* < 0.0001

**Table 3:** CPC negative men who did not comply with the criteria of active surveillance.

	Total PSA	Free percent PSA	Gleason score	Number of cores positive for cancer	Percent of core infiltrated with cancer
Patient number 1	7.8 ng/mL	7%	7	2	30
Patient number 2	4.52 ng/mL	15%	6	5	40
Patient number 3	4.68 ng/mL	24%	7	1	5
Patient number 4	4.71 ng/mL	11%	6	4	4
